# Supplementary figures and images for: Transcriptome analysis of sevoflurane exposure effects at the different brain regions
Source: PLoS One. 2020 Dec 15;15(12):e0236771. doi: 10.1371/journal.pone.0236771 (PMC7737892; doi:10.1371/journal.pone.0236771)

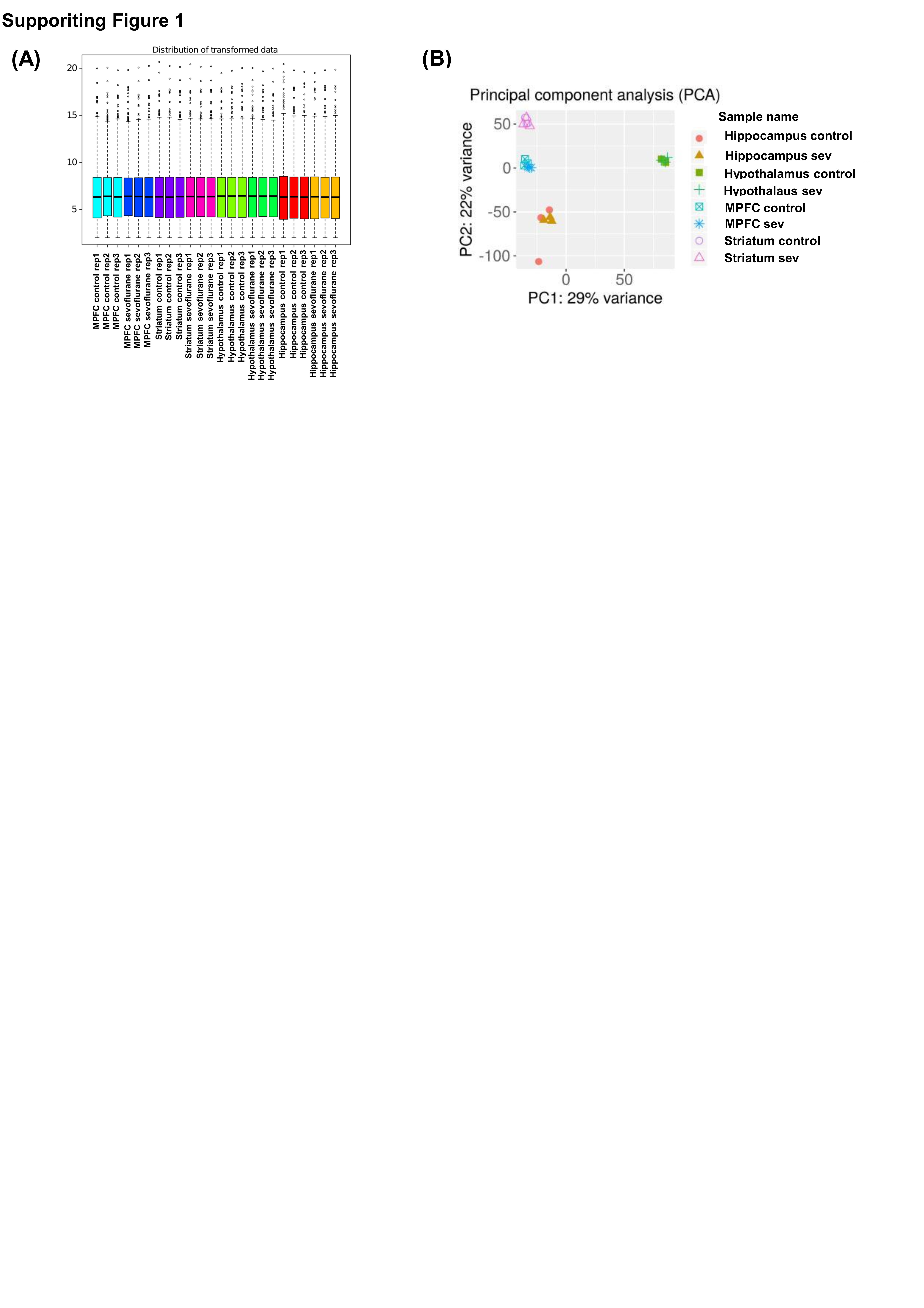

Supplement: S1 Fig — (A)The distribution of log2 ((count per million) +4) after normalization. (B)PCA-plot for RNA-seq data. (TIF) [file pone.0236771.s001.tif]

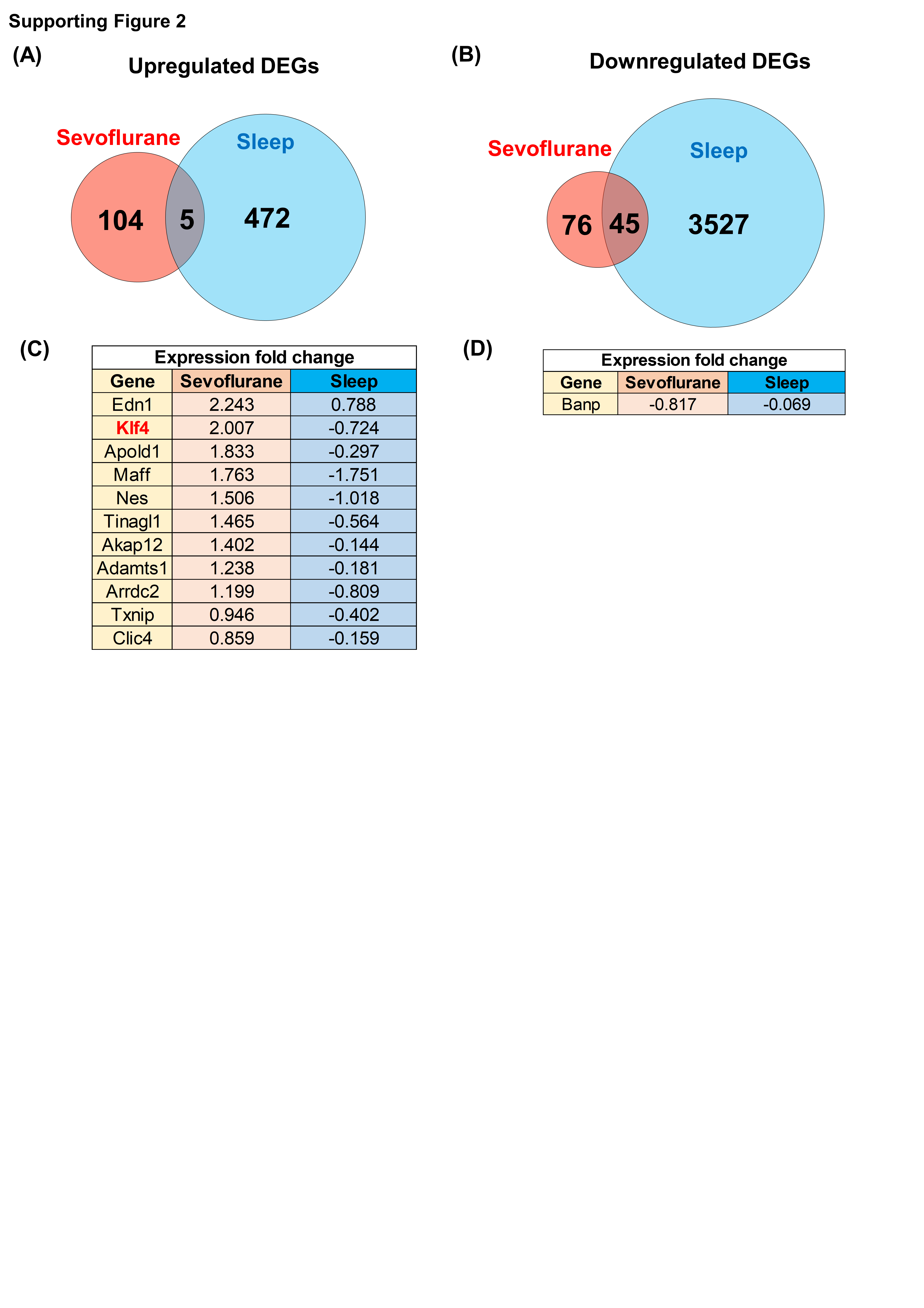

Supplement: S2 Fig — (A, B) DEGs were extracted from the transcriptome array data of the cortical cortices of the sleeping mice. The DEGs in the medial prefrontal cortex of the mice that inhaled sevoflurane and those in the cortical cortices of the sleeping mice were compared. The Venn-diagrams for the upregulated (A) and downregulated DEGs (B) are shown. (C) Table of the expression fold change (log2) of the genes commonly upregulated in the four parts of the brain of the mice that inhaled sevoflurane. (D) Table of expression fold changes (log2) of the genes commonly downregulated in the four parts of brain of the mice that inhaled sevoflurane. (TIF) [file pone.0236771.s002.tif]

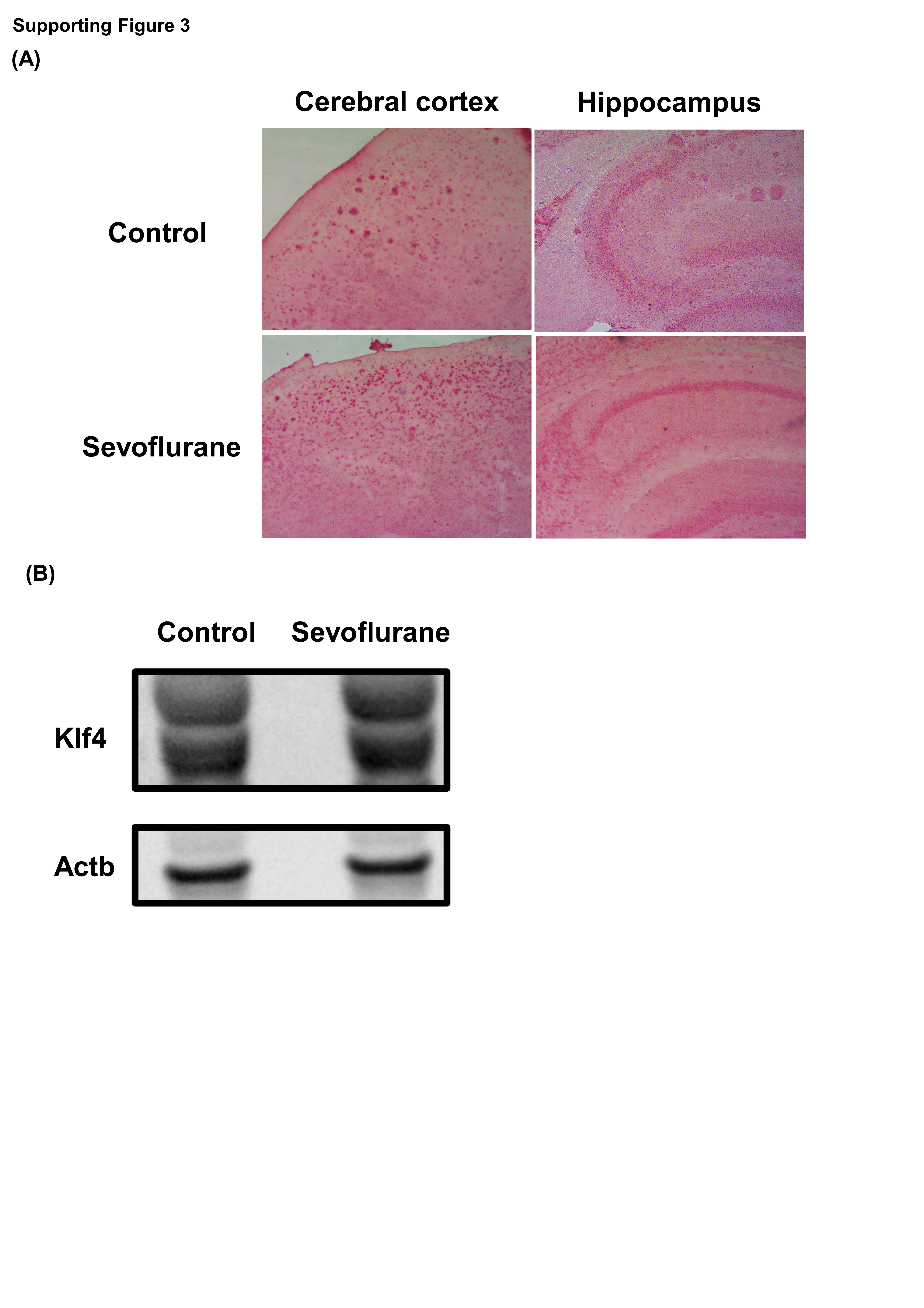

Supplement: S3 Fig — Representative image of immunohistochemical analysis of KLF4 for cerebral cortex and hippocampus of mice exposed to sevoflurane. Western blotting for hippocampus of brains exposed to sevoflurane. (TIF) [file pone.0236771.s003.tif]
